# Efficacy comparison between cryoablation and radiofrequency ablation for patients with cavotricuspid valve isthmus dependent atrial flutter: a meta-analysis

**DOI:** 10.1038/srep10910

**Published:** 2015-06-03

**Authors:** Yi-He Chen, Hui Lin, Cheng-Long Xie, Xiao-Ting Zhang, Yi-Gang Li

**Affiliations:** 1 Department of Cardiology, Xinhua Hospital affiliated to the Medical School of Shanghai Jiaotong University, 1665 Kongjiang Road, Shanghai, 200092, China; 2 Department of Respiratory, the second affiliated hospital of Wenzhou Medical University, Wenzhou 325027, China; 3 Department of Neurology, Xinhua Hospital affiliated to the Medical School of Shanghai Jiaotong University, 1665 Kongjiang Road, Shanghai, 200092, China; 4 Department of Dermatology, the second affiliated hospital of Wenzhou Medical University, Wenzhou 325027, China

## Abstract

We perform this meta-analysis to compare the efficacy and safety of cryoablation versus radiofrequency ablation for patients with cavotricuspid valve isthmus dependent atrial flutter. By searching EMBASE, MEDLINE, PubMed and Cochrane electronic databases from March 1986 to September 2014, 7 randomized clinical trials were included. Acute (risk ratio[RR]: 0.93; *P* = 0.14) and long-term (RR: 0.94; *P* = 0.08) success rate were slightly lower in cryoablation group than in radiofrequency ablation group, but the difference was not statistically significant. Additionally, the fluoroscopy time was nonsignificantly reduced (weighted mean difference[WMD]: −2.83; *P* = 0.29), whereas procedure time was significantly longer (WMD: 25.95; *P* = 0.01) in cryoablation group compared with radiofrequency ablation group. Furthermore, Pain perception during the catheter ablation was substantially less in cryoabaltion group than in radiofrequency ablation group (standardized mean difference[SMD]: −2.36; *P* < 0.00001). Thus, our meta-analysis demonstrated that cryoablation and radiofrequency ablation produce comparable acute and long-term success rate for patients with cavotricuspid valve isthmus dependent atrial flutter. Meanwhile, cryoablation ablation tends to reduce the fluoroscopy time and significantly reduce pain perception in cost of significantly prolonged procedure time.

Atrial flutter is a common atrial arrhythmia due to macroreentrant propagating counter-clockwise or clockwise through the cavotricuspid valve isthmus^1^. There are estimated 200,000 new atrial flutter patients each year in USA^2^, and there is close interrelationship between atrial flutter and atrial fibrillation^3^. Patients with atrial flutter usually experience significant symptoms (palpitation, breathlessness, chest discomfort and fatigue), furthermore, it may cause serious severe effects including thromboembolic events, myocardial ischemia, congestive heart failure, and in rare may lead to tachycardia mediated cardiomyopathy. Catheter ablation of cavotricuspid valve isthmus is nowadays the first-line nonpharmacological treatment for atrial flutter, and the acute success rate is high and the recurrence rate during the follow-up is low when bidirectional conduction block (BCB) is achieved^4^. Although radiofrequency is the common energy source for ablation procedures, it can injure the adjacent cardiac tissue and cause steam pops or thrombus, which may sometimes result in coronary artery occlusion, myocardial perforation, cardiac tamponade or stroke^5^,^6^.

Cryoablation, an alternative energy source, has been reported to have similar acute and long term efficacy and safety rates as radiofrequency ablation for patients with atrial arrhythmias from randomized or nonrandomized clinical studies^7^. In addition, it may overcome some disadvantages of radiofrequency ablation, especially the severe chest discomfort, and longer fluoroscopy time than cryoablation^8^. Thus, cryoablation ablation has challenged the predominant status of radiofrequency ablation in atrial flutter treatment strategy. So far, there were only limited published randomized controlled trials comparing cryoablation versus radiofrequency ablation for atrial flutter with small patient number which make the presented conclusion less credible.

There was already meta-analysis comparing cryoablation with radiofrequency ablation in patients with atrial flutter^9^, recently, newly published randomized controlled trails (RCTs) provided more valuable information on this issue. In this meta-analysis, we included 7 RCTs comparing the efficacy and safety between cryoablation and radiofrequency ablation in patients with atrial flutter, in an effort to supply updated information on this issue.

## Results

### Baseline characteristics and risk bias of included studies

**Figure 1:**
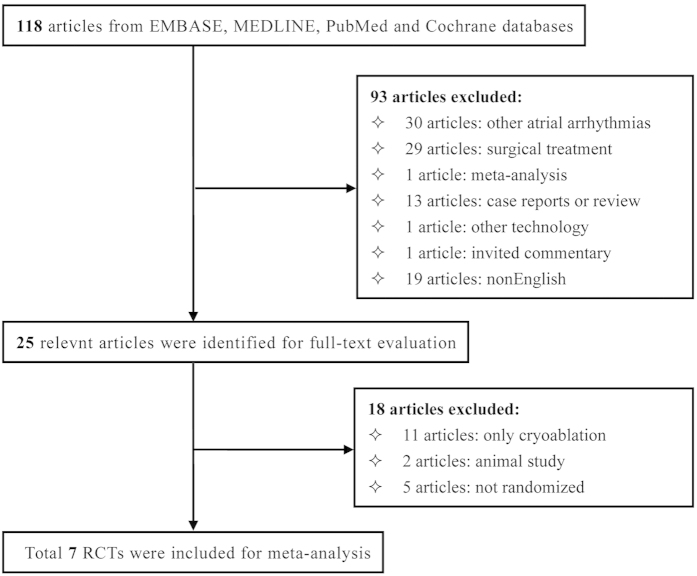
Flow diagram of included studies.

The baseline characteristics of the 7 included studies were summarized in Table 1. Our initial literature search identified 118 articles, after title and abstract screening, 93 articles were excluded. Full text review was performed in the remaining 25 articles. Finally, 7 studies, which enrolled 496 patients (83.5% male, mean age 63.1 years, mean follow-up 10.0 months), 247 patients referred for cryoablation and 247 patients referred for radiofrequency ablation procedure, were included in this meta-analysis (Fig. 1). Five studies were performed in Europe, 1 in Oceania and 1 in China. All but two studies adopted 8-mm-tip cryoablation with the nadir temperature of approximately −80 °C. Radiofrenquency ablation was conducted by using 8-mm-tip in six studies or irrigated tip in two studies with the maximum ablation energy and temperature range from 30–80 W and 40–70 °C, respectively. History of atrial fibrillation referred for each treatment (43.4% for cryoablation and 41.2% for radiofrequency ablation) was similar between two groups. Additionally, the score of risk bias of eligible studies ranged from 3 to 4 points, which showed a mild to moderate risk of bias in this meta-analysis.

### Acute success rate of BCB

**Figure 2:**
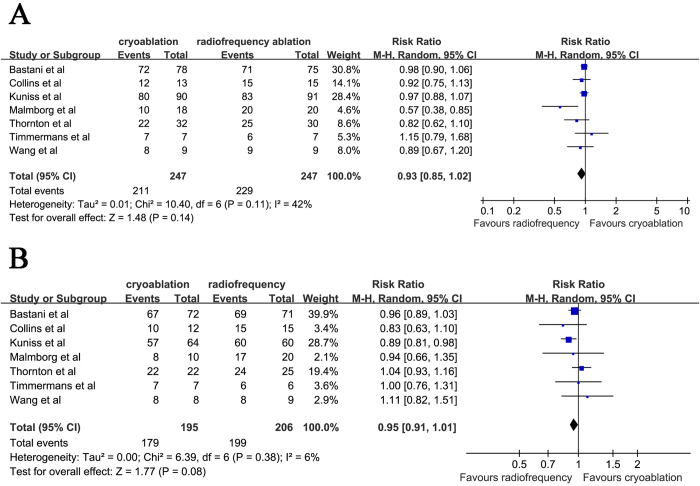
Forest plot of acute success of BCB (**A**) and long-term success (**B**) in patients referred for cryoablation or radiofrequency ablation. BCB, bidirectional conduction block.

BCB was immediately achieved in 211 of 247 patients (85.4%) in cryoablation group and in 229 of 247 patients (92.7%) in radiofrequency ablation group. Acute success rate of BCB was slightly lower in cryoablation group compared with radiofrequency ablation group but the difference was not statistical significant (RR: 0.93 [95% CI: 0.85 to 1.02]; *P* = 0.14; heterogeneity *I*^2^ = 42%; *P* = 0.11) (Fig. 2A). The funnel plot was symmetric by visual inspection which indicated there was no publication bias (data not shown).

### Long-term free from atrial flutter post ablation

Analysis of the long-term success during the follow-up, free from atrial flutter was reported in 179 out of 195 patients in cryoablation group (91.8%) and 199 out of 206 patients in radiofrequency group (96.6%). The long-term success rate was also slightly lower in cryoablation group than radiofrequency ablation group though without statistically difference (RR: 0.95 [95% CI: 0.91 to 1.01]; *P* = 0.08; heterogeneity *I*^2^ = 6%; *P* = 0.38) (Fig. 2B).

### Procedure time and Fluoroscopy time

**Figure 3:**
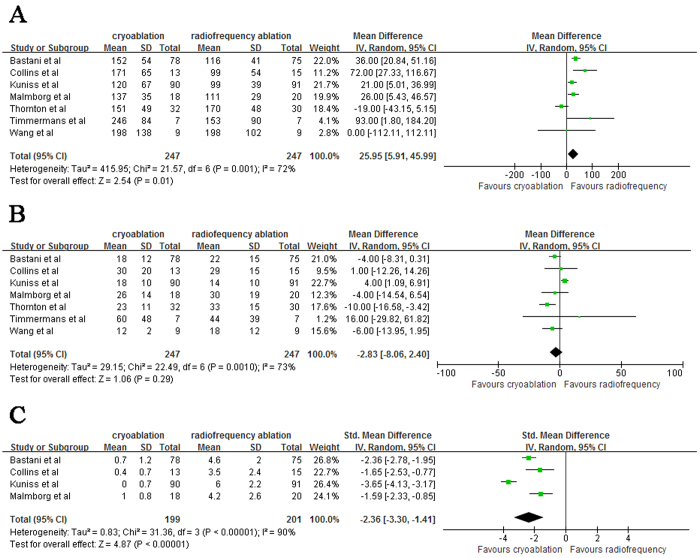
Forest plot of procedure time (**A**), fluoroscopy time (**B**) and pain perception (**C**) in patients referred for cryoablation or radiofrequency ablation.

Cryoablation was associated with a longer total procedure time compared to radiofrequency ablation (WMD: 25.95 [95% CI: 5.91 to 45.99]; *P* = 0.01; heterogeneity *I*^2^ = 72%; *P* = 0.01) (Fig. 3A). The fluoroscopy time was nonsignificantly reduced in cryoablation group compared with radiofrequency ablation group (WMD: −2.83 [95% CI: −8.06 to 2.40]; *P* = 0.29; heterogeneity *I*^2^ = 73%; *P* = 0.001) (Fig. 3B).

### Pain perception

Five out of 7 studies evaluated pain perception according to visual analogue scale (VAS), and the study reported by Timmermans *et al.*^11^ was excluded since mean pain score during the procedure was not reported in this study. In addition, score of VAS ranged from 0 to 10 in three studies with the remainder ranged from 0 to 100, thus the pooled estimates of pain perception from four eligible studies was expressed by SMD and accompanying 95% CI. Cryoablation, compared with radiofrequency ablation, significantly reduced the pain score during the procedure (SMD: −2.36 [95% CI: −3.30 to −1.41]; *P* < 0.00001; heterogeneity *I*^2^ = 90%; *P* < 0.00001) (Fig. 3C).

## Discussion

This meta-analysis including 7 randomized controlled studies indicated that both cryoablation and radiofrequency ablation were safe and effective for treating patients with cavotricuspid valve isthmus dependent atrial flutter. Pooled estimates presented a slight tendency towards cryoablation for lower acute success rate of BCB and long-term success for atrial flutter free, but the difference was not statistically significant. Additionally, patients referred for cryoablation experienced longer procedure time compared with radiofrequency ablation but the fluoroscopy time was slightly shorter. Notably, patients suffered from significantly more pains estimated by the VAS scale during the radiofrequency ablation compared to cryoablation ablation procedure.

Cavotricuspid valve isthmus dependent atrial flutter could usually be terminated by antiarrhythmic drug or catheter ablation^8^,^12^. In view of the side effect of long term drug intake and the increasingly important role of radiofrequency ablation, cavotricuspid valve isthmus ablation is recommended as a standard therapy for atrial flutter now^4^,^13^. Even though radiofrequency ablation is widely applied in treatment for atrial or ventricular arrhythmias, procedure-related severe complications such as coronary artery occlusion or cardiac tamponade need to be considered carefully for clinical decision making^5^,^6^,^14^,^15^. As a reasonably alternative energy source, cryoablation had not only been demonstrated to be an effective approach but also minimized the risk of cardiovascular events^7^,^16^. In a large 160 patients pivotal study conducted by Feld and his colleagues, 87.5% patients referred for cryoablation achieved acute success and 80.3% patients, who completed 6 months follow-up, maintained sinus rhythm^17^. Moreover, another single center study enrolled 180 patients also showed high acute and chronic success rate which were identical to radiofrequency ablation^18^. Furthermore, Perez *et al.*^19^ performed a meta-analysis including 158 studies, indicated that cryoablation yielded a similar success rate to radiofrequency ablation with different catheter types. Taken together, abovementioned results from the clinical studies strongly suggest that cryoablation might be a promising and effective alternative approach for atrial flutter treatment.

Nevertheless, there was a trend for slightly lower acute and long-term success rate for patients underwent cryoablation ablation compared with radiofrequency ablation. Following factors might be responsible for this phenomenon: cryoablation ablation was applied by using focal “point by point” method, whereas radiofrequency ablation created consistent ablation lines by using “drag and burn” method across the cavotricuspid valve isthmus^8^,^20^, additionally, physiological reversibility of cryoablation was possible without enough ablation time or lower enough temperature, myocardium may recover from freezing injury^21^.

This analysis showed that total procedure time was longer for cryoablation compared with radiofrequency ablation. It might be explained by the longer learning curve of cryoablation ablation, and longer cryothermal energy delivery time, and, each cryothermal energy delivery lasts for 240 seconds in most of clinical studies^20^,^22^,^23^. From the technical view, the characteristic of firmly adherance to the myocardial tissue during the cryoablation procedure could result in strong catheter stability, which could thus prevent the operators and patients from longer X-ray exposing. Pooled estimates indicated that there was a slight tendency towards cryoablation for shorter fluoroscopy time, however, the difference was not statistical significant. Nevertheless, we believed that with the enlargement of sample size, accumulation of more operational experience, and adjustment of other confounders, cryoablation’s advantage over radiofrequency ablation in fluoroscopy time would become more obvious.

It is noteworthy that radiofrequency ablation caused significant more severe pain than cryoablation ablation during the procedure, which thus be responsible for the higher sedative or analgesic drug intake in patients underwent radiofrequency ablation^20^,^23^,^24^,^25^. More importantly, pain related position change or catheter movement might also increase the risk for complications. So far, the mechanism of pain associated with radiofrequency ablation remained unknown. It was possible that excessive thermal energy delivery could stimulate the cardiac autonomic nerves adjacent to cavotricuspid valve isthmus, and rarely the pain might be linked to procedure-induced coronary artery injury or pericarditis^26^.

Although there was already similar meta-analysis on this topic before, it was the last update including important new data and provided more reliable evidence. Compared with the meta-analysis performed by Andrew *et al.*^9^, present work added an newly published study, which was a prospective, single-blinded, randomized and controlled single-centre trial with high quality score and markedly enlarged the sample size as well. Moreover, beside the main endpoints, this meta-analysis also emphasized the patient tolerability. It was another important information, not only caused the use of analgesic and sedative drug, but also influenced the decision making during the ablation procedure. Furthermore, Perez *et al.*^19^ performed a meta-analysis including 158 studies and evaluated the long-term outcomes after ablation of atrial flutter with any catheter types. The pooled results, however, might overestimate the efficacy and safety of catheter ablation because of the large amount of non-RCTs. Thus, the present meta-analysis made up for the deficiency by including only RCTs and provided stronger evidence base for clinical recommendation.

### Study limitation

First, despite the fact that all randomized controlled studies including RCTs published during recent years were included in this meta-analysis, however, the total number of patients was only 494, which is still not large enough to draw solid precise conclusion regarding the efficacy and safety of cryoablation versus radiofrequency ablation for patients with cavotricuspid valve isthmus dependent atrial flutter. Second, variation in ablation parameters (the size of catheter tip, temperature and time of ablation procedure) may also have potential influence on the efficacy of these two approaches. Third, due to the different follow-up duration and lack of intensive monitoring of atrial arrhythmias occurrence, the true recurrence of atrial flutter may be underestimated. Finally, there was significant heterogeneity among some studies which may influence the statistical validity of this meta-analysis, thus conclusion had to be drawn with a great caution under these circumstances. Large, randomized controlled studies with rigorous design should be conducted to assess the efficacy and safety between these two procedures in the future.

## Conclusion

In patients with cavotricuspid valve isthmus dependent atrial flutter, cryoablation and radiofrequency ablation produce comparable acute success rate of BCB and long-term success of atrial flutter free. Moreover, slightly shorter fluoroscopy time and less pain perception during the catheter ablation is balanced by longer procedure time for patients underwent cryoablation ablation compared with radiofrequency ablation. With the accumulation of experience and clinical data from randomized controlled studies as well as technological advance, cryoablation procedure might increasingly present its advantages over radiofrequency ablation and become a promising alternative approach in the near future for treating patients with cavotricuspid valve isthmus dependent atrial flutter.

## Methods

### Search strategy

EMBASE, MEDLINE, PubMed and Cochrane electronic databases were searched for articles published from March 1986 to September 2014 by using the following medical subject terms and keywords: “atrial flutter” and “ablation”, together with the following search terms “cryoablation” or “cryothermal” or “cryoballoon” or “cryocatheter”. No language restriction was enforced in the search strategy. Reference lists from the resulting publications and reviews were used to identify further relevant articles.

### Study selection

All relevant articles were reviewed by two independent investigators (Yi-He Chen and Hui Lin) to assess their eligibility for analysis, with discrepancies solved by consensus. Study for inclusion should met the following criteria: a) studies were RCTs; b) without prior history of ablation for atrial flutter or atrial fibrillation; c) cryoablation versus radiofrequency ablation; d) report of acute success of bidirectional conduction block (BCB), recurrence, procedure time and fluoroscopy time. Exclusion criteria were: a) Non-RCTs; b) case reports, abstracts, comments, reviews, and editorials.

### Data extraction and quality assessment

From each eligible study, data extraction and quality assessment were independently conducted by two investigators (Chen-Long Xie and Xiao-Ting Zhang). Discrepancies were resolved by consensus or by discussion with another investigator. The following data were collected from each study: surname of the first author, publication year, and number of patients referred for cryoablation and radiofrequency ablation, % male gender, the type of catheter and parameters of ablation, history of atrial fibrillation and the follow-up duration, as well as the primary outcomes. Efficacy was assessed by acute success rate of BCB and long-term success (which expressed as patients free from recurrence), the safety outcome was defined as procedure time, fluoroscopy time and pain perception during the catheter ablation. If the included studies lacked important information, we contacted the corresponding author by e-mail. The risk bias of each study was assessed by using the method recommended by Cochrane Handbook for Systematic Reviews including the following criteria: randomization method, allocation concealment, and method of blind, incomplete outcome data, selective outcome reporting and other sources of bias.

### Data analysis

We calculated relative risks (RRs) and accompanying 95% confidence interval (CI) for dichotomous variables (acute success rate of BCB, recurrence of atrial flutter) from each study. Continuous variables were expressed as weighted mean difference (WMD) and accompanying 95% CI when outcome measurements in all studies are made on the same scale. On the contrary, standardized mean difference (SMD) was utilized to assess the same outcome but measured in a variety of ways^10^. To be conservative, overall estimates were pooled and compared with a random-effects model. Heterogeneity of pooled outcomes in different studies was assessed by Cochran Q statistic and inconsistency index (*I*^2^) statistic. Two tailed *P* value <0.05 was set as statistically significant for all estimates of effect except for test of heterogeneity (*P* < 0.1). Publication bias was assessed by visual inspection of a funnel plot. All data analysis was completed with Review Manager software (RevMan, The Cochrane Collaboration, Oxford, England, version 5.3).

## Additional Information

**How to cite this article**: Chen, Y.-H. *et al.* Efficacy comparison between cryoablation and radiofrequency ablation for patients with cavotricuspid valve isthmus dependent atrial flutter: a meta-analysis. *Sci. Rep.* **5**, 10910; doi: 10.1038/srep10910 (2015).

## Figures and Tables

**Table 1:** The baseline characteristics of included studies.

**Author**	**Year**	**Data Source**	**Mean Age (year)**	**Male Gender (%)**	**No. Of Patients**			**Ablation Type**		**No. Of AF**		**Follow-Up duriation (month)**	**Primary Outcomes**	**Quality Score**
					**Cryo**	**RF**	**Total**	**Cryo**	**RF**	**Cryo**	**RF**			
Bastani *et al.*^23^	2013	Sweden	65.0	85.0	78	75	153	8-mm-tip −80 °C	3.5-mm-irrig-tip 30–40 W,40–42 °C	43	35	6	1. Acute success; 2. Recurrence; 3. Procedure time; 4. Fluoroscopy time; 5. Pain perception	4
Kuniss *et al.*^20^	2009	Germany	65.5	80.7	90	91	181	8-mm-tip NA	8-mm-tip 50–80 W,55–60 °C	23	28	3	1. Acute success; 2. Recurrence; 3. Procedure time; 4. Fluoroscopy time; 5. Pain perception	3
Malmborg *et al.*^25^	2009	Sweden	58.7	87.5	20	20	40	8-mm-tip NA	8-mm-tip 65 W,60 °C	11	8	15.1	1. Acute success; 2. Recurrence; 3. Procedure time; 4. Fluoroscopy time; 5. Pain perception	3
Thornton *et al.*^27^	2008	Netherlands	56.0	88.7	32	30	62	8-mm-tip −75 °C	8-mm-tip 60 W,60 °C	25	22	3	1. Acute success; 2. Recurrence; 3. Procedure time; 4. Fluoroscopy time;	3
Wang fang *et al.*^24^	2007	China	59.6	94.4	9	9	18	6-mm-tip NA	4-mm-irrig-tip 30 W,55 °C	2	3	22	1. Acute success; 2. Recurrence; 3. Procedure time; 4. Fluoroscopy time;	3
Collins *et al.*^28^	2006	Australia	64.9	71.4	13	15	28	8-mm-tip −75 to −80 °C	8-mm-tip 50–80 W,60 °C	1	4	14.7	1. Acute success; 2. Recurrence; 3. Procedure time; 4. Fluoroscopy time; 5. Pain perception	4
Timmermans *et al.*^11^	2003	Netherlands	55.0	78.6	7	7	14	6-mm-tip NA	8-mm-tip 55 W,70 °C	NA	NA	6	1. Acute success; 2. Recurrence; 3. Procedure time; 4. Fluoroscopy time; 5. Pain perception	3

Cryo: cryoablation; RF: radiofrequency ablation; irrig: using irrigated tip; W: watt; AF: atrial fibrillation; NA: not available
